# 

**Published:** 1919-11-22

**Authors:** 


### PASSING EVENTS AND TOPICS.

### Mr. Curry's Assailants Sentenced.

Vincent and Anda, the two men who were charged with breaking into the Elysée Restaurant, and also with assaulting Mr. Curry, the Clerk to the Governors of Guy's Hospital, have been found guilty and sentenced at the Central Criminal Court, the first to eighteen months' imprisonment with hard labour, and the second to twelve months.

### St. Thomas's Hospital.

Mr. G. D. C. Tracey, of Emmanuel College, Cambridge, has been awarded the University Entrance Scholarship for 1919-20 at St. Thomas's Hospital Medical School.

### Enquiry Into the Use of Native Medicines.

We understand that under the auspices of the Royal Society an anthropological expedition is at present at work in Uganda. The expedition has been equipped and sent out, thanks to the generosity of Mr. P. J. Mackie, and a collection of all sorts of native medicines is being made. As soon as these are brought back to England they will be submitted in duplicate for examination to the Royal Society and to the University of Edinburgh. It is hoped that information regarding new drugs may be secured, as many of the most valuable remedies used in this country owe their origin to primitive workers and healers. In any case, valuable knowledge of native customs and methods is sure to be obtained.

### The New Registrar of Births and Deaths.

The Minister of Health is now to be responsible for the whole administration of the Department of the Registrar-General of Births, Deaths, and Marriages, including the administration of the census. To facilitate the carrying into effect of the altered constitutional position created by this decision, the Minister has appointed Mr. S. P. Vivian, an Assistant Secretary in the Ministry, to be Deputy Registrar-General of Births, Deaths, and Marriages.

### Pony Friend of a Hospital.

A Shetland pony, which has been the means of raising close on £500 for the King Edward VII. Hospital, Cardiff, has been presented to that institution. Sir William James Thomas and Lady Thomas were present, and the former handed in a cheque for £1,000 to endow a bed as a thank-offering for the recent birth of their son and heir.

### Doctor Leaves Legacy to V.C.

Dr. Guthrie Rankin, of Harley Street, left estate valued at £54,362. Amongst several specific legacies was one of £500 to "his friend" Lieut.-General Sir William Babtie, V.C., and one of the same amount to his brother, Mr. F. G. Rankin, "who was last heard of as resident in South Africa." Dr. Rankin left £1,000 each to the Samaritan Funds of the Seamen's Hospital, Greenwich, and the Royal Waterloo Hospital for Women and Children, at which hospitals he was honorary physician.

### Profiteering and Patent Medicines.

The Tottenham Profiteering Committee decided that a complaint against Messrs. Boots. Ltd., had been established. The complainant stated that he had bought from the firm a bottle of Fellowe's syrup, for which he was charged 8s. 6d., and on removing the label discovered that the original price, 5s., had been three times altered to 6s. 6d., 7s 5d., and 8s. 6d. Messrs. Boots were ordered to refund 3s. 6d., and notice of appeal was given.

### Matrons' and Sisters' Appointments.

County Mental Asylum, Isle of Wight.—Miss E. J. Thompson has been appointed assistant matron. She was trained at the Prestwich County Asylum, Lancs, and has been assistant matron at Palmerston House, Co. Dublin; also at Hendon Grove Private Asylum. She has recently been doing maternity work in London, and holds certificates of the Central Midwives Board and of the Royal Sanitary Institute.

General Hospital, Merthyr Tydfil.—Miss M. Gibbs Bowen has been appointed assistant matron. She received her training at the General and Eye Hospital, Swansea.

Isolation Hospital, Fareham, Hants.—Miss E. Howard has been appointed matron. She was trained at the Poplar and Stepney Infirmary and at the Royal Chest Hospital, City Road. She has held appointments as ward sister at Kingston Hospital, Bristol City Infectious Hospital, St. Pancras Infirmary, and matron at the Isolation Hospital, Chester-le-Street. She has worked in Leeds in the T.F.N.S. Miss Howard is a member of the College of Nursing.

Monkwearmouth and Southwick Hospital, Sunderland.—Miss C. K. Wallace has been appointed matron. She was trained at the Royal Victoria Infirmary, Newcastle-on-Tyne, and has been matron of the 3rd Durham Temporary Hospital, Sunderland.

Municipal Maternity Hospital, Leicester.—Miss Hilda Mason and Miss E. Wood have been appointed matron and ward sister. Miss Mason was trained at Stapleton Infirmary, Bristol. She has been maternity sister at the Nightingale Home, Derby, and at Aberdeen, and has also done military service at home and abroad in the T.F.N.S. Miss Wood was trained at the Clayton Infirmary, Bradford, has done private maternity work, and has seen military service under the T.F.N.S.

Norwich Municipal Authority.—Miss G. Corder, R.R.C., has been appointed health visitor. She was trained at East London Hospital for Children, Shadwell, and Guy's Hospital, London. She has held appointments of sister at the Civil Hospital, Kandy, Ceylon; assistant matron, Colonial Hospital, Trinidad, B.W.I.; as sister and acting matron in Q.A.I.M.N.S.R. on active service abroad and hospital ship. Miss Corder has been twice mentioned in despatches.

Queen Alexandra's Military Nursing Service for India.—Miss E. M. Bell and Miss W. M. Thomson have been appointed nursing sisters.

Southport Babies' Home.—Miss B. Baxter has been appointed head sister. She was trained at the Victoria Hospital, Mansfield, and has since been temporary sister at Leigh Road Infirmary, Leigh, and done private nursing in Southport. She took her qualification in midwifery at St. Mary's Hospital, Manchester.

Union Infirmary, Dudley.—Miss E. M. Parker has been appointed night sister. She was trained at the Birmingham Union Infirmary, has been staff nurse under the Stone Joint Hospital Board. She has also done military service under the T.F.N.S. at the 1st Northern General, Liverpool.

Westmoreland Sanatorium (for Men), near Grange-over-Sands, Lancs.—Miss A. Dobie has been appointed sister. She was trained at the Royal Alexandra Infirmary, Paisley, where she became acting ward sister. She has also been staff nurse, Norfolk War Hospital, Norwich; staff nurse, Bury St. Edmunds General Hospital, and the Consumptive Sanatorium, Bridge of Weir.

### Personalia.

G. Hardyman, M.B., F.R.C.S., has been appointed hon. consulting surgeon of the Royal Mineral Water Hospital, Bath.

Mr. L. Billington has been appointed to the post of dispenser at the St. Mary's, Islington, Infirmary, Highgate Hill, London. Mr. Billington is a qualified pharmacist, by examination of the Pharmaceutical Society of Great Britain.

### The College of Nursing
### (Limited by Guarantee).

EAST LANCASHIRE CENTRE (MANCHESTER BRANCH).

The general meeting of the Manchester Branch was held at the Manchester Royal Infirmary on the 12th inst., and Mrs. Jessop Hulton, the Hon. Secretary of the Nation's Fund for Nurses, gave a very interesting lecture on the present divorce laws.

At the next meeting of the Manchester Branch, which will be held on Wednesday, the 26th inst., at the Royal Infirmary, Manchester, Miss Sparshott, C.B.E., R.R.C., will give a lecture (with lantern slides) on her recent visit to Norway.

LIVERPOOL CENTRE.

Monday, November 24, 7 p.m.—A members' meeting will be held in the University Lecture Theatre, on the first floor of the New Arts building. Dr. J. E. Nevins will lecture on "Four Years in an Indian Court," illustrated by lantern slides.

BIRMINGHAM CENTRE.

Mrs. Martin Harvey gave an interesting address at Metchley House (by kind invitation of Mrs. Byng Kenrick) on November 5 at a meeting on behalf of the Nation's Tribute Fund for Nurses. The chair was taken by Mr. Leonard Gamgee, F.R.C.S., and Miss Musson, R.R.C., also spoke on the work of the College.

The next meeting of the Centre will be on
**Tuesday, December 2, at 4 p.m.**
Tea will be provided, and a lantern lecture by Dr. Ratcliffe on "Places of Interest in Belgium" will follow.

Members are reminded that it is important that any change of name or address should be notified to the Hon. Secretary of the local Centre.

### Hospital Gala at Brighton.

A gala on a large scale was held on Thursday, October 30, at Brighton in aid of the Royal Sussex County Hospital, the Royal Alexandra Hospital, and the new Sussex Hospital for Women. The management of the Palace Pier gave generous assistance, and something like a record in early matinees was held in the theatre at 11 o'clock in the morning, at which there was a crowded house, as also at the performance in the afternoon. A number of well-known artistes gave their services, among them being Mr. George Robey, Miss Ellaline Terris, and Mr. and Mrs. Seymour Hicks. The Pier was crowded all day, and a most successful effort was concluded in the evening by a series of boxing matches, during which the colours worn by Jimmy Wilde in some of his recent contests were put up for sale, and realised some £23 for the funds.

### The Hove Creche Fund.

A PUBLIC-SPIRITED MAYOR.

The town of Hove, Brighton, has shown interest in the Baby Week movement, during which the necessity of a crèche was confirmed. Thanks to the Mayor, it will soon be an accomplished fact. Alderman A. R. Sargeant was Mayor of Hove during the whole of the war, and in recognition of his work from 1914 to 1919 the townspeople presented him on the 7th instant with gifts in kind and money. The money consisted of the balance of the fund, and at the presentation the Alderman proposed to make the £300 a nucleus of the Hove Crèche Fund.

